# Exploring Emerging Challenges: Survey on Phlebotomine Sand Flies and *Leishmania infantum* at the Northern Endemic Border in Europe

**DOI:** 10.3390/pathogens13121074

**Published:** 2024-12-07

**Authors:** Damiana Ravasi, Manuela Schnyder, Valeria Guidi, Tim Haye, Diego Parrondo Monton, Eleonora Flacio

**Affiliations:** 1 Institute of Microbiology, Department for Environment Constructions and Design, University of Applied Sciences and Arts of Southern Switzerland (SUPSI), 6850 Mendrisio, Switzerland; valeria.guidi@supsi.ch (V.G.); diego.parrondo@supsi.ch (D.P.M.); eleonora.flacio@supsi.ch (E.F.); 2 Institute of Parasitology, University of Zurich, 8057 Zurich, Switzerland; manuela.schnyder@uzh.ch; 3 CABI, 2800 Delémont, Switzerland; t.haye@cabi.org

**Keywords:** *Leishmania infantum*, phlebotomine sand flies, Switzerland, *Phlebotomus*, canine leishmaniosis

## Abstract

Although Switzerland is currently not endemic for canine leishmaniosis (CanL), imported cases of this emerging zoonosis are regularly detected. Also, phlebotomine sand flies, vectors of the causative agent *Leishmania infantum*, are present in the southern part of the country, in Canton Ticino, and endemic foci of CanL have been recently described in neighboring Italian municipalities. In 2022 and 2023, we evaluated the distribution of phlebotomine sand flies and the presence of antibodies against *L. infantum* in domestic dogs and cats in Ticino and Mesolcina (Canton of Grisons). An entomological survey was also carried out in the northwest of Switzerland (cantons Basel-Stadt and Basel-Landschaft) close to an area in Germany where potential vectors are present. No sand flies were caught there. In Ticino, 15 out of 20 sites surveyed (75%) were positive for phlebotomine sand flies. *Phlebotomus perniciosus*, a potential vector of *L. infantum*, was the most abundant species, with site densities ranging from 0.1 to 5.3. The parasite was not detected in females. *Leishmania infantum* seroprevalences of 3.0% and 1.6% were observed in 101 and 126 dog and cat sera, respectively. Although, at this time, the risk of endemic CanL is extremely low, integrated surveillance and prevention measures should be considered.

## 1. Introduction

Leishmaniasis is a tropical disease caused by parasitic protozoa of the genus *Leishmania* (Kinetoplastida: Trypanosomatidae) and transmitted to vertebrates through the bites of phlebotomine sand flies (Diptera: Phlebotominae) [1]. This neglected disease is endemic in tropical and subtropical climates, including all regions bordering the Mediterranean Sea in southern Europe, where it is mainly associated with the species *L. infantum* [2,3]. *Leishmania infantum* causes zoonotic cutaneous (CL) and visceral leishmaniasis (VL) in humans. CL manifests as skin lesions, often self-healing within a few months but leaving disfiguring scars. VL causes systemic disease, affecting several internal organs, usually the spleen, liver and bone marrow, and is fatal if left untreated [3,4,5]. Currently, no effective vaccines for the prevention of leishmaniasis are available, and the best way to prevent infection is the application of topical insect repellents for protection against sand fly bites [2].

Dogs are highly susceptible to *L. infantum* infections and are considered the main reservoir host for human VL in European endemic areas [6,7,8]. Canine leishmaniosis (CanL) can, at high cost and effort, be treated, but the parasite is never completely eliminated [9]. Asymptomatic and treated dogs can still function as reservoirs for the transmission of the parasites to sand flies [10,11]. Recent studies also suggested domestic cats as primary and/or secondary reservoir hosts contributing to zoonotic transmission [12,13,14,15,16]. Other mammals implicated as reservoirs are foxes, badgers, equids, rodents and lagomorphs [6].

In central Europe, cases of autochthonous human leishmaniasis are currently very rare, although the lack of mandatory notification of clinical cases in most European countries makes it difficult to assess the real situation [17,18]. Nevertheless, the risk of leishmaniasis emergence in these non-endemic regions is being increased by drivers such as the northward expansion or increased densities of phlebotomine vectors (through climate change causing milder winters and warmer summer temperatures), the spread of parasites (through traveling and importation of infected animals) and the increased exposure to vectors (through urbanization of endemic rural areas and the opposite, through increase of green spaces in urban areas) [19,20]. Italy has seen a 30-year northward expansion and increase in density of *L. infantum* vectors *Phlebotomus perniciosus* and *Ph. neglectus*, which are now present in the northern continental regions and are associated with the occurrence of autochthonous CanL and human cases [21,22,23,24]. Similar patterns have been documented in Spain and France [25,26]. In addition, the increased traveling with or translocation of dogs from endemic to non-endemic regions has led to an increase in CanL cases in central and northern Europe, to the point that it has become one of the most frequently diagnosed travel diseases [21,27,28,29,30].

In Switzerland, low densities of *Ph. perniciosus*, the most competent vector for *L. infantum*, have been repeatedly recorded since 1944 in the southern part of the country, in Canton Ticino [31,32,33,34,35]. Other species recorded in Canton Ticino are *Ph. neglectus*, another vector of *L. infantum* that is only sporadically detected, *Ph. mascittii*, a suspected but unproven vector of *L. infantum* [36] and *Sergentomyia minuta*, known to mainly feed on reptiles [31,32,33,34,35]. A retrospective survey of 545 sera collected between 2010 and 2016 from owned dogs in Canton Ticino showed an *L. infantum* seroprevalence of 2.9%. All positive dogs originated from or had visited endemic areas [34].

Canton Ticino is, therefore, still considered non-endemic for CanL. However, its proximity (even within 10 km), along with the high similarity in vegetation and climate, to neighboring areas in Italy in which foci of endemic CanL have been recently described [21,37], represents a potential risk for the emergence of CanL and, accordingly, the transmission of *L. infantum* to humans. Consequently, regular assessment of the epidemiological risk is essential. In 2022 and 2023, we reassessed the distribution of phlebotomine sand flies in Canton Ticino and carried out in parallel epidemiological surveys on the presence of *L. infantum* in domestic dogs and cats. An entomological survey was also carried out in the northwest of Switzerland, in the cantons of Basel-Stadt and Basel-Landschaft, as *Ph. mascittii* has been recorded since 1999 just across the border in southwest Germany (Upper Rhine Valley) [38], and climatic conditions seem to be suitable also for *Ph. perniciosus* in these regions [39,40].

## 2. Materials and Methods

### 2.1. Study Areas

**Figure 1:**
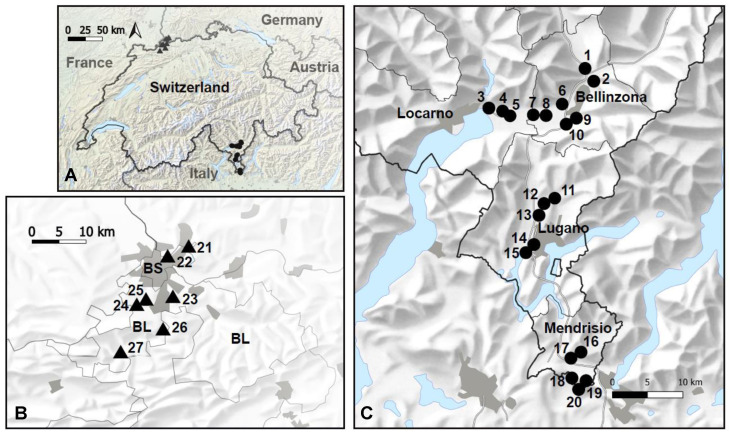
(**A**) Phlebotomine sand flies were surveyed in seven sites around Basel (triangles) and in 20 sites in Canton Ticino (circles). (**B**) Trapping sites in the cantons of Basel-Stadt and Basel-Landschaft. (**C**) Trapping sites in Canton Ticino. Maps from the Federal Office of Topography swisstopo modified in qGIS 3.10.10.

The main survey of phlebotomine sand flies was conducted in Canton Ticino, which is one of the three large southern Alpine cantons of Switzerland, along with Valais and the Grisons and borders the Italian regions of Piedmont to the southwest and Lombardy to the southeast. A significant portion (the northern and central parts) of Canton Ticino is part of the Lepontine Alps and presents geomorphological and environmental characteristics considered unsuitable for colonization by phlebotomine sand flies. The trapping effort focused, therefore, on the territory that is part of the Lombard Prealps (Figure 1), where phlebotomine sand flies have been previously recorded. The Lombard Prealps extend from the southern areas of Canton Ticino to the northern areas of Lombardy and are characterized by foothills and typical components of agriculture (including the presence of habitats, such as vineyards, favorable to phlebotomine sand flies) next to residential, industrial and commercial urbanized areas. The climate of this area is strongly affected by the Mediterranean Sea, with warm and moist summers and mild winters.

A smaller survey was carried out north of the Alps in the two adjacent cantons of Basel-Stadt and Basel-Landschaft. These are in the northwest of Switzerland and on the border with France and Germany. Compared to Canton Ticino, they are characterized by a cooler, more continental climate, with colder winters and less humidity in summer. Their landscape is characterized by gently rolling, forested hills, agricultural land and urbanized areas.

### 2.2. Entomological Surveys

In Canton Ticino, the survey of phlebotomine sand flies was conducted along a north–south transect from the northern districts of Locarno and Bellinzona to the southern districts of Lugano and Mendrisio (Figure 1). Twenty trapping sites were selected in areas potentially suitable for sand flies: south-facing valleys and hills, at an altitude ranging from 200 to 567 m.a.s.l., in peri-urban habitat, and close to humans and major domestic potential hosts of *L. infantum* (i.e., dogs). The sites included farms, cracks in roadside walls, private chicken coops, dog pensions, stables, campsites and dog walks (Table 1).

In Basel-Stadt, the survey was carried out in the municipality of Riehen, just northeast of the city of Basel and close to the area in Germany where sand flies have been observed. In Basel-Landschaft, the survey was carried out along a north–south transect in the districts of Arlesheim and Laufen (Figure 1). The seven trapping sites included farms and areas where private owners breed either rabbits or chickens (Table 1).

The main sampling was carried out in 2022 during the summer months (from the second half of June to the first half of August) when the highest phlebotomine sand fly densities are expected [35,41]. At each sampling site, we positioned 10 to 20 sticky traps made from 20 × 20 cm castor oiled paper (total area: 0.4 to 0.8 m^2^) and one CO_2_-baited CDC light trap (BG-Pro, Biogents, Regensburg Germany). In three locations around Basel, only sticky traps were used (Table 1). The sticky traps were replaced once per week, and the CO_2_-baited CDC light traps were run for one night every week. The survey was replicated in 2023 in the five sites (i.e., sites 16–20) in Canton Ticino, closest to the border with Italy, in the Mendrisio district (Figure 1), using only CO_2_-baited CDC light traps.

Phlebotomine sand flies caught in sticky traps were detached using a fine brush, placed in 96% ethanol and stored individually at 4 °C in 1.5 mL reagent tubes. Sand flies caught with CO_2_-baited CDC light traps were stored individually at −80 °C in 1.5 mL reagent tubes. Specimens were identified at the species level by DNA barcoding. DNA was isolated from the entire insect with a QIAamp^®^ DNA Mini Kit (Qiagen, Hilden, Germany), according to the manufacturer’s instructions. The cytochrome c oxidase I (COI) gene was amplified using standard protocols and primers for insect DNA barcoding, namely the forward primer LCO1490 (5′-GGTCAACAAATCATAAAGATATTGG-3′) and the reverse primer HCO2198 (5′-TAAACTTCAGGGTGACCAAAAATCA-3′) [42]. PCR amplifications were performed in 25 μL containing 1× Taq PCR Master Mix Kit (Qiagen) and primers (0.3 μM each; Microsynth, Balgach, Switzerland). Conditions were one cycle at 94 °C for 10 min; 40 cycles of 94 °C for 1 min, 1 min at 52 °C, 72 °C for 1 min; and a final extension at 72 °C for 7 min. PCR products were purified using Sephadex^®^ G-100 (Sigma-Aldrich, Buchs, Switzerland) according to the manufacturer’s instructions and sent to Microsynth for sequencing. Raw sequences were analyzed in BioNumerics v.7.00 software (Applied Maths, Sint-Martens-Latem, Belgium). The obtained sequences were compared with *Phlebotomus* spp. sequences deposited in GenBank (https://www.ncbi.nlm.nih.gov/nucleotide/, accessed on 1 October 2024). Molecular identification was first tested using two *Ph. perniciosus* adults, kindly provided by the Instituto de Salud Carlos III (ISCIII), Madrid, Spain. The COI sequences obtained from the phlebotomine sand flies analyzed in this study have been deposited in GenBank under the accession numbers PQ496044–PQ496218.

Sand fly density was calculated as the number of specimens collected divided by the sampling effort [43,44]. The sampling effort is the number of trapping nights multiplied by the trap area (m^2^) for sticky traps and by the number of traps for CO_2_-baited CDC light traps.

### 2.3. Detection of Leishmania infantum in Phlebotomine Sand Flies

The detection of *L. infantum* was performed by real-time PCR targeting a 123 bp region of the kinetoplast DNA minicircle (kDNA minicircle), using primers and probe previously described [45,46]. In general, assays designed on kinetoplast DNA minicircle allow sensitive detection of the parasite due to its high copy number (thousands of copies per parasite [47,48]), but identification is possible only at the genus or subgenus level [48]. This protocol was successfully adopted for the detection of *L. infantum* in phlebotomine sand flies in southern Italy [49].

DNA extracts used for the species identification of sand flies were analyzed for the presence of *L. infantum*. Real-time PCR assays targeting the *L. infantum* kDNA minicircle were performed in a 7500 Fast Real-Time PCR System (Applied Biosystems, Waltham, MA, USA), with the following thermal cycling conditions: 2 min at 50 °C, 20 s at 95 °C, followed by 40 cycles at 95 °C for 3 s and 60 °C for 30 s. Each reaction consisted of a 20 μL solution containing 10 μL TaqMan^®^ Fast Advanced PCR Master Mix (Applied Biosystems), 0.9 μM of LEISH-1 (5′-AACTTTTCTGGTCCTCCGGGTAG-3′) and LEISH-2 (5′-ACCCCCAGTTTCCCGCC-3′) primers, 0.2 μM of TaqMan-MGB probe (FAM-5′-AAAAATGGGTGCAGAAAT-3′-MGBEQ), 1 μL 10× Exo IPC mix, 0.2 μL 50× Exo IPC DNA (TaqMan^®^ Exogenous Internal Positive Control Reagents, Applied Biosystems), and 2 μL of template DNA. Samples were tested in duplicates, and each amplification run included no template control samples and positive samples consisting of genomic DNA from *L. infantum* JPCM5 promastigotes.

### 2.4. Surveys of Domestic Hosts

In Switzerland, every dog must be identifiable with a microchip and is registered in a national database. Accordingly, stray dogs are not present, and every dog has an owner. In September 2023, the number of registered dogs in Switzerland was 561,209, and 34,478 dogs had been imported in 2022 (data from Identitas AG, https://tierstatistik.identitas.ch/en/, accessed on 18 October 2023). The number of registered dogs in Canton Ticino in September 2023 was 34,255 (6.1% of the number of registered dogs in Switzerland). A field serosurvey was conducted through veterinary practices/practitioners covering all eight districts of Canton Ticino and the Mesolcina area in the Canton of Grisons. Blood samples from different categories of dogs (imported, clinically suspect, healthy) were collected by veterinary practices as part of the standard diagnostic procedures. Dog owners participated voluntarily in the screening of their dogs, and samples were collected with written owner consent. Of the 17 practices addressed, nine practices in total (six in the northern part of Canton Ticino and Mesolcina and three in the southern part of Canton Ticino, close to the Italian border) submitted canine blood samples. Identification of samples positive to *L. infantum* was based on serological detection of antibodies through an established and validated ELISA method: in vitro cultivated promastigote stages of *L. infantum* (zymodeme MON1) were used as antigens and combined with an anti-dog IgG (gamma chain specific) conjugate, as previously described [29]. The test was defined with a sensitivity of 100% (95% Confidence Intervals—CI: 85.5–100% in asymptomatic dogs, 88.4–100% in symptomatic dogs) and a specificity of 96% (95% CI: 86.3–99.5%).

For cats, since validated serological tests for the detection of antibodies in these animals are barely available, a novel test was developed in 2022 within this study [50]. Starting from the ELISA established for dogs [29], we substituted the anti-dog conjugate with an anti-cat-IgG conjugate and evaluated the optimal concentrations of all components in checkerboard titrations. Two validated samples from Spain were used as positive controls. Overall, 307 cat sera from north of the Alps without suspicion of *L. infantum* infection were used to evaluate sensitivity (estimated at 99.3%), and 58 sera of cats with other proven parasitic infections were used to evaluate cross-reactions: of these, 3 were above the threshold (single cats infected with *Isospora* sp., *Toxocara cati* and taeniids, respectively), indicating high specificity of the ELISA (98%, 95% CI: 97.4–98.7%). Anticipating that further evaluation is required, we have adopted this ELISA for retrospective and prospective epidemiological investigations. For the retrospective analysis, we used 126 cat sera previously collected in Canton Ticino; no information regarding clinical signs nor traveling history was available. For the prospective analysis, the same veterinary practices/practitioners previously contacted for dogs were asked for cat sera. We obtained 13 cat sera, for which limited data regarding the animal were delivered. These cats were virtually all asymptomatic; one cat was known to originate from Italy. In September 2023, there were 733,255 cats registered in Switzerland, of which 3.4% (25,051 cats) were registered in Canton Ticino. There are no registration requirements for cats by law, and it is estimated that approximately 1.863 million cats are present in the country.

## 3. Results

### 3.1. Phlebotomine Sand Flies

Surveys of phlebotomine sand flies in northwest Switzerland (cantons Basel-Stadt and Basel-Landschaft) were carried out from 28 June until 2 August 2022. No sand flies were caught. Surveys in Canton Ticino were carried out from 17 June until 9 August 2022, and phlebotomine sand flies were caught during the whole trapping period. In the five sites in Mendrisio district close to the Italian border, CO_2_-baited CDC light traps were activated one extra week on 16 August 2022; no sand flies were caught then. The same five sites in Mendrisio district were also surveyed in 2023, from 5 June until 16 August, but with CO_2_-baited CDC light traps only. In that year, phlebotomine sand flies were caught from mid-June until the end of the trapping period.

In 2022, fifteen (75%) out of 20 trapping sites in Canton Ticino were positive for sand flies. This included all five sites in Mendrisio district. In 2023, four of the five sites surveyed in Mendrisio district were positive. Out of 97 sand flies collected in 2022, the majority (94%) were caught with CO_2_-baited CDC light traps, while only six specimens were caught with sticky traps. Consequently, sticky traps were not used in the survey in 2023, and overall density calculations were based only on catches from CO_2_-baited CDC light traps. In general, more males (62 and 74 in 2022 and 2023, respectively) were caught than females (35 and 13 in 2022 and 2023, respectively).

**Figure 2:**
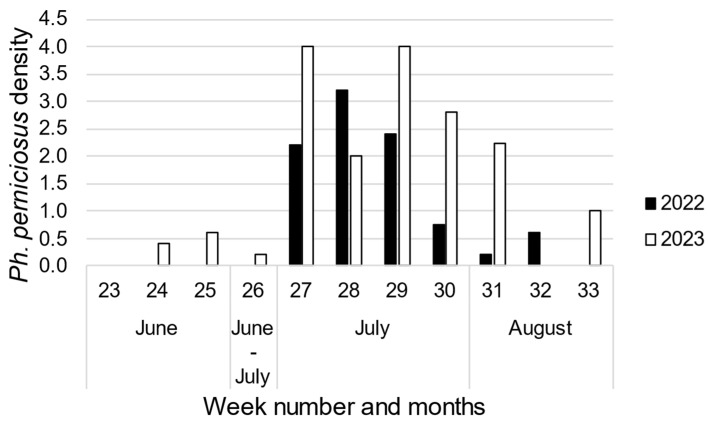
Seasonal trend of *Ph. perniciosus* in southern Canton Ticino in 2022 and 2023.

Four species of phlebotomine sand flies were collected. Most specimens belonged to the species *Ph. perniciosus* (62 and 83 specimens in 2022 and 2023, respectively), followed by *S. minuta* (18 and 3 specimens), *Ph. mascittii* (10 and 1) and *Ph. neglectus* (1 specimen caught in 2022). In both 2022 and 2023, the highest densities of *Ph. perniciosus* in the five sites in the Mendrisio district were observed in July (Figure 2). It must be noted, however, that CO_2_-baited CDC light traps were activated only in one week (i.e., week 25) in June 2022. The densities of the other three species were too low to identify a trend in the season.

**Figure 3:**
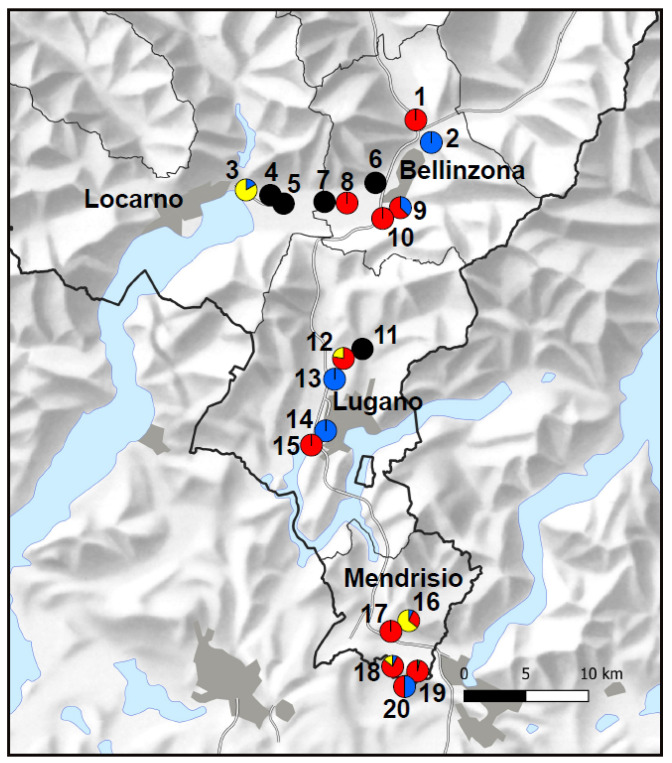
Spatial distribution of phlebotomine sand flies in Canton Ticino. The colors show the percentage of each sand fly species caught in each site in 2022. Blue: *Ph. mascittii*; green: *Ph. neglectus*; red: *Ph. perniciosus*; yellow: *S. minuta*; black: no sand flies. Maps from the Federal Office of Topography swisstopo modified in qGIS 3.10.10.

The 2022 horizontal spatial distribution of the four species of phlebotomine sand flies in Canton Ticino is shown in Figure 3.

More specimens of phlebotomine sand flies (n = 61) were caught in the five Mendrisio sites in 2022 than in the other 15 sites (n = 30) investigated. *Phlebotomus mascittii* was found in eight sites located in all four districts surveyed. Densities were slightly higher at the northernmost sites (Table 2). *Phlebotomus perniciosus* seemed to follow an opposite distribution, with higher densities in the southernmost surveyed sites in the Mendrisio district. *Phlebotomus neglectus* was found only at one site, in the Mendrisio district. *Sergentomyia minuta* was found at four sites and did not seem to show a pattern in horizontal spatial distribution and densities. The altitudes of positive sites varied between 207 and 447 m.a.s.l. The densities of sand flies did not seem to be influenced by the type of site or proximity to a particular potential host, such as domestic dogs (Table 2).

### 3.2. Leishmania infantum in Phlebotomine Sand Flies

In 2022, we tested DNA extracts from a total of 36 adult female phlebotomine sand flies from Canton Ticino, and none were positive for *L. infantum*. As for 2023, a total of 14 DNA extracts from female sand flies collected in Mendrisio district were analyzed, all of which proved negative for *L. infantum*.

### 3.3. Leishmania infantum in Domestic Hosts

From September 2022 to September 2023, 101 dog sera from nine veterinary practices were analyzed for antibodies against *L. infantum* by ELISA (Table 3). The dogs were originally from the following countries: Italy (n = 59 dogs), Switzerland (n = 13), Spain (n = 6), Portugal (n = 3), France (n = 2), Romania (n = 2) and Croatia (n = 1). For six dogs, the origin was unknown, and for nine dogs, no information was delivered. Three samples were seropositive, and one was defined as ‘questionable’ (meaning that the optical density was in an intermediate range for which the repetition of the test earliest after two months would be recommended). All four dogs had been imported from Italy. Clinical signs were present in all three seropositive dogs, with one dog being already under allopurinol treatment, one dog showing apathia, and the third dog presenting apathia and nasal ulcera. The dog was defined as ‘questionable’ and asymptomatic. This resulted in a seroprevalence of 3.0%, with a 95% CI of 0.6–8.4% (Table 3). The results obtained were extrapolated to the overall dog population in Canton Ticino using AMICUS (https://www.amicus.ch, accessed on 1 October 2023), the national database for the registration of dogs in Switzerland (data retrieved from Identitas AG, https://tierstatistik.identitas.ch/en/, accessed on 18 October 2023). In September 2023, 34,255 dogs were registered in Canton Ticino and 561,209 in Switzerland as a whole. Therefore, extrapolated from the obtained seroprevalence, this would result in 1028 positive dogs in Canton Ticino, with a broad range of 206–2877.

Of the 126 cat sera tested in the retrospective survey, two samples (1.6%, 95% CI: 0.2–5.6%) tested positive for *L. infantum* antibodies (Table 3). The results obtained were extrapolated to the overall cat population in Canton Ticino using Anis (https://www.anis.ch, accessed on 1 October 2023), the national database for the registration of cats in Switzerland (data retrieved from Identitas AG, https://tierstatistik.identitas.ch/en/, accessed on 18 October 2023). In September 2023, 25,051 cats were registered in Canton Ticino and 733,255 in Switzerland as a whole. Therefore, extrapolated from the obtained prevalence, this results in 401 potentially positive cats, with a range of 50–1403. Of the 13 cat sera prospectively obtained from the collaborating veterinary practices in 2023, one from a cat without anamnestic data was positive for *L. infantum*.

## 4. Discussion

In 2022 and 2023, we investigated the presence of phlebotomine sand flies in southern (Canton Ticino) and northwestern (cantons Basel-Stadt and Basel-Landschaft) Switzerland. In parallel, we conducted a serosurvey on *L. infantum* in domestic dogs and cats in Canton Ticino.

The entomological survey carried out in 2022 around Basel was probably the first in this area of Switzerland, except for one location surveyed in 2011 in the district of Laufen (canton Basel-Landschaft) with a negative outcome [35]. No sand flies were caught in the seven sites surveyed in 2022. The presence of phlebotomine sand fly *Ph. mascittii* has been documented several times, the most recent being in 2018 in areas of southwest Germany (i.e., Baden-Wuerttemberg) bordering Basel [38,51]. *Phlebotomus mascittii* is a suspected vector of *L. infantum,* but its vector competence has not been conclusively clarified so far [36,52]. The number of *Ph. mascittii* caught in southwestern Germany has always been low so far (less than 10 individuals caught per trap session [38]). *Phlebotomus perniciosus*, considered the most important competent vector for *L. infantum* in Europe, was also recorded in southwest Germany but only once in 2001, and further north from Baden-Wuerttemberg, in the Rhineland-Palatinate [30,38]. Given that climatic suitability studies predict favorable conditions for both sand fly species in the coming decades in the northern and northeastern parts of Switzerland on the border with France and Germany [39], surveys in the region of Basel should be carried out in the future.

In the surveys carried out in 2022 and 2023 in Canton Ticino, we recorded the same four species (i.e., *Ph. mascittii*, *Ph. neglectus*, *Ph. perniciosus* and *Sergentomyia minuta*) of phlebotomine sand flies that have already been documented in the last 15 years [34,35] and further back in time [32,33]. *Phlebotomus neglectus* is also a competent vector for *L. infantum*, although seemingly less important than *Ph. perniciosus*. *Sergentomyia minuta* is a herpetophilic sand fly and the natural vector of *Leishmania (Sauroleishmania) tarentolae* in reptiles. Although its role in the transmission of *L. infantum* does not appear to be important [53], a study in Portugal did detect the parasite in this species of sand fly [54].

Both in 2022 and 2023, the highest numbers of phlebotomine sand flies were observed in the first three weeks of July, indicating this period was the seasonal population peak in Canton Ticino, as observed at northern Italian latitudes [21,55]. The use of two different trapping methods led to different outcomes. The number (i.e., 91) of sand flies caught in 2022 using CO_2_-baited CDC light traps (an attractive method) was much higher than the number (i.e., 6) caught by sticky traps (an interception method). These results support previous findings that attractive traps are more effective than non-attractive traps for collecting sand flies in areas where their abundance is low, as seems to be the case in Canton Ticino [56]. The number of *Ph. perniciosus* females (i.e., 9) and sand fly females in general (i.e., 36) caught in 2022 was lower than the number of males (i.e., 56 *Ph. perniciosus* males and 62 sand fly males in general). This seems to disagree with previous observations of *Ph. perniciosus* females being highly phototropic [57,58] and thus highly attracted by light traps [56]. Specific tests should be carried out to verify the attraction of *Ph. perniciosus* males and females to different trap settings in the specific conditions present in Canton Ticino.

Of the 20 sites surveyed in Canton Ticino in 2022, more sites positive for *Ph. perniciosus* and the other sand fly species were recorded in the southern districts (Lugano and Mendrisio) of the canton, compared to the northern districts (Bellinzona and Locarno). The densities of *Ph. perniciosus* ranged from 0.1 to 5.3 (average: 1.2) and were, in general, higher in the Mendrisio district. The highest density (4.1 in 2022 and 5.3 in 2023) was recorded at one site located very close (<1 km) to the Italian border. Catches obtained in the five sites in Mendrisio district in 2023 were similar to 2022. The densities of *Ph. mascittii* (range 0.1–0.4) and *S. minuta* (range 0.3–1.1) were, in general, low. *Phlebotomus mascittii* was caught at seemingly slightly higher densities in the Bellinzona and Locarno districts. *Sergentomyia minuta* did not show a clear pattern in horizontal distribution, and *Phlebotomus neglectus* was only recorded once at one site in Mendrisio district, with a density of 0.1.

Comparisons with the older surveys carried out in multiple municipalities in Ticino in 1981–1983, 2009 and 2010 [33,34,35] are problematic due to different methods used to calculate the densities or to represent them. For instance, in 1981, Knechtli and Jenni [33] obtained high densities of sand flies (up to 12.5 for *Ph. perniciosus*) with sticky traps. However, the densities were expressed per trap area (m^2^), while in the following studies, the trap area was additionally multiplied by the number of trapping nights to better take into account the sampling effort. The last survey of phlebotomine sand flies in Canton Ticino was conducted in 2016. On that occasion, seven sites were sampled at intervals of eight–nine days on average from the end of June to the end of August using sticky traps [34,35]. *Phlebotomus perniciosus* was only recorded in three sites (in the districts of Mendrisio, Lugano and Bellinzona), with densities ranging between 0.1 and 0.2, therefore similar or lower to the densities recorded in 2022 and 2023. *Phlebotomus mascittii* was recorded in six out of seven sites (districts of Mendrisio, Lugano, Bellinzona and Locarno), with densities varying between 0.04 and 1 [34,35], which were also in the same order of magnitude as the densities recorded in the 2022 survey.

The observed seroprevalence for *L. infantum* antibodies in dogs in Canton Ticino (3.0%, 95% CI: 0.6–8.4%) was very similar to the seroprevalence of 2.9% observed between 2010 and 2016 in 545 sera collected from owned dogs in the same area [34]. In both surveys, all positive dogs (of known origin) were imported from endemic countries. For comparison, the seroprevalence of CanL in endemic areas can exceed 30% [59]. In our study, *L. infantum* was not detected in the caught female sand flies, a result that can be expected when the incidence of infection with CanL is low [23]. Extrapolating the obtained seroprevalence from the limited number of analyzed dogs, compared to the overall dog population in Canton Ticino, this results in 1028 positive dogs, with a wide range of uncertainty (206–2877). With the assumption that dogs imported to the other areas of Switzerland may originate from the same countries as the dogs imported into Canton Ticino (which could represent a bias with Ticino bordering Italy, but unfortunately, the national database only gives the countries of import for the whole country, and not broken down by cantons), the extrapolation indicates 1414 *Leishmania*-positive dogs imported into Switzerland in 2022, with a possible variation from 310 to 3965. Importantly, dogs are frequently imported from *Leishmania* endemic countries, mostly through animal welfare organizations and in respect of the current legislation: there is no restriction for importing *Leishmania*-positive dogs to Switzerland or Germany, and there is even systematic dog import from endemic countries [29]. For comparison, more than 100,000 *Leishmania*-positive dogs may be already present in Germany, according to recent estimates [60]. However, the risk of human infection is still expected to be extremely low in this country due to the limited presence of suitable vectors. Another large German survey (with indirect and direct methods) confirmed a substantial risk that traveling dogs, typically returning from Spain, Italy and France, may import *L. infantum* to central Europe [61]. A fairly high percentage of traveling dogs resident in central Europe may already be *Leishmania*-positive [62,63] and act as reservoir hosts of *L. infantum* [64] but may not have been identified due to incubation periods of up to 9 years [29]. Furthermore, to evaluate the risk of endemic establishment and autochthonous transmission, together with the number of infected reservoir hosts and the presence of a significant number of vectors, further factors (e.g., a variety of environmental factors) need to be considered [65].

Related to the reservoir host spectrum, despite the limited knowledge of epidemiological and clinical aspects, as well as the management of feline leishmaniosis [14], Asfaram et al. [12] suggested cats as primary and/or secondary reservoir hosts (and therefore ‘cryptic’), contributing to the transmission of *Leishmania* spp. to humans and dogs. Still, there is a need for consolidated evidence-based knowledge, as there may be important differences between dogs and cats associated with transmission, pathogenesis and best management practices [15]. *Leishmania* strains identified in cats displayed the same biological properties as canine and human strains [66]. Further studies from Spain and Italy focused on risk factors and clinical aspects of feline leishmaniosis: comorbidities such as FIV (Feline Immunodeficiency Virus) may increase the onset of dermatological and other lesions [13,16], while most infected cats may be asymptomatic and immunocompetent and were, therefore, described as ‘cryptic reservoirs hosts’ [12]. Consequently, it is relevant to test cats and adopt diagnostic techniques to identify infected cats. A comparative analysis of 180 stray cats showed good agreements between serological procedures (ELISA or IFAT) for the detection of antibodies or with PCR performed on blood samples [67]. The analysis of lymph node aspirates may be more sensitive; however, conjunctival swabs are also discussed as a less invasive procedure that represents a potential alternative [68]. In the presented retrospective analysis, 1.6% of cats (CI: 0.2–5.6%) from Ticino were seropositive for *Leishmania* antibodies, and one of the few prospectively analyzed cats was seropositive. In an extrapolation with the number of registered cats in Ticino (with a clear bias towards under-registering cats), approximately four hundred cats may be infected. Compared to dogs, cats are less frequently imported and are taken on vacation less often, but considering the widespread presence of free-roaming cats and their closeness to humans, further knowledge on the potential transmission of *Leishmania* to cats and their role in the establishment of endemic areas is warranted.

Canton Ticino borders the Italian regions of Piedmont to the southwest and Lombardy to the southeast. Both of these regions have experienced the spread of *Ph. perniciosus* and the emergence of autochthonous CanL in recent years. In the Piedmont region, the first stable foci of CanL were reported between 1999 and 2001 [69]. At that time, seroprevalence in resident dogs was 4.5% in Turin and 5.8% in Ivrea, which is located about 50 km north-east of Turin. In the same period, densities of *Ph. perniciosus* were 10.6 individuals/m^2^ sticky traps in Turin and 7.3 in Ivrea at the peak of the activity season [69]. Later, in 2007, densities of *Ph. perniciosus* in newly endemic areas of human VL in Piedmont ranged from 12 to 18 individuals/m^2^ sticky traps, whereas the prevalence of *L. infantum* in dogs was 40.35% (CI 95% 37.01–43.78%) [55]. Less information is available for the Lombardy region: in a survey carried out in 2021, 670 sand flies were caught, with *Ph. perniciosus* being the most abundant species (87.76%), followed by *Ph. perfiliewii* (7.31%) and *Ph. neglectus* (3.13%) [37]. Sadly, densities were not specified.

In Canton Ticino, based on the serosurveys performed in this study and the previous one [34], we have not seen a large increase in seroprevalence of *L. infantum* in dogs over the years, as observed in Piedmont. Foci of CanL has not yet been reported, and cases of human leishmaniasis have, to our knowledge, not been described. The density of phlebotomine sand flies, including that of the competent vector *Ph. perniciosus*, seems to be still low compared to the neighboring Italian areas. However, finding sand flies over several years at the same trapping sites indicates population stability at these sites. Concurrently infected dogs are present, and more infected dogs are very likely regularly imported from endemic regions. Therefore, the risk of autochthonous transmission is present, and the epidemiological situation might change in the coming years. Overall, the results from this study, in parallel to the general situation observable in Switzerland’s neighboring countries, advocate for integrated preventive and control measures encompassing human health (e.g., sharing epidemiological data with human medicine and collaborating in an adjustment of the legislation as well as recommendations for the implementation in practice), animal health (e.g., early diagnosis and treatment of CanL cases, both imported and autochthonous; larger monitoring of dogs, cats and other vertebrate reservoirs of *L. infantum*), the vectors (e.g., surveying geographical distribution, biology and density of the vectors, with particular interest in areas with CanL cases and possible foci; definition of sentinel areas for regular monitoring; molecular detection of *L. infantum* in sand flies) and their shared environment.

## 5. Conclusions

According to the survey carried out in 2022 and 2023, the abundance of *Ph. perniciosus* and other phlebotomine sand flies in Canton Ticino seems to be relatively low and patched. This, coupled with the absence of *L. infantum* in the phlebotomine females collected, suggests that the risk of phlebotomine-borne leishmaniasis is still low in this region of Switzerland. However, the presence of dogs infected by *L. infantum* is a concern. Infected dogs are mainly imported rather than traveling animals, and their number is very likely underestimated in view of their lifelong infection despite treatments. This, with the presence of phlebotomine sand flies competent for *L. infantum* transmission in Canton Ticino, in addition to the presence of endemic foci of CanL in Italian areas close to the border with this canton, indicates that continuous surveillance of the distribution and density of sand flies should be considered in the very near future. Furthermore, it will be important to continue promoting appropriate prophylactic measures (i.e., pyrethroid-containing spot-on solutions or collars for dogs or fluralaner-containing products) for all dogs in Canton Ticino and Mesolcina against phlebotomine bites during the whole vector season. With pyrethroids being toxic for cats, other preventative measures, such as keeping cats indoors from dusk to dawn, are indicated. Although not evaluated, imidacloprid- or fluralener-containing products may also be useful for preventing phlebotomine bites in cats (preventative measures for cats reviewed in [70]). The role of cats as popular domestic animals and susceptible hosts for *L. infantum*, with an often subclinical course of the infection, should not be underestimated. For dogs already known to be infected with *L. infantum*, we strongly recommend prescribing the use of efficacious drugs to prevent phlebotomine bites during the vector season, with the aim to impede the infection of local phlebotomine and, therefore, the potential transmission to other hosts. In this regard, other measures, such as controlled imports with compulsory *Leishmania* testing, should be discussed.

## Figures and Tables

**Table 1:** Overview of sampling sites in the cantons Ticino (TI), Basel-Stadt (BS) and Basel-Landschaft (BL). Districts: Bellinzona (BEL), Locarno (LOC), Lugano (LUG) and Mendrisio (MEN). LT: light trap; ST: sticky trap.

Site	Canton (District)	Latitude, Longitude	Altitude (m.a.s.l.)	Site Type	Potential Domestic Hosts	Trap Types
1	TI (BEL)	46.22649, 9.02952	237	Dog shelter	Dogs	LT, ST
2	TI (BEL)	46.21237, 9.0387	241	Campsite	Dogs	LT, ST
3	TI (LOC)	46.17893, 8.85174	218	Private house	Chickens, guinea pigs, dogs	LT, ST
4	TI (LOC)	46.17731, 8.86896	200	Horse stable	Horse, dogs	LT, ST
5	TI (LOC)	46.17628, 8.87016	200	Chicken farm	Chickens, dogs	LT, ST
6	TI (BEL)	46.18121, 8.9836	216	Farm	Cattle, dogs, chickens	LT, ST
7	TI (BEL)	46.17102, 8.93746	220	Woodland path	Dogs, horses	LT, ST
8	TI (BEL)	46.16783, 8.95076	207	Farm	Rabbits, chickens, cats	LT, ST
9	TI (BEL)	46.16375, 9.00529	256	Private breeder	Rabbits, chickens, cats	LT, ST
10	TI (BEL)	46.16144, 8.99828	225	Horse stable	Horses, cats, dogs	LT, ST
11	TI (LUG)	46.06026, 8.9614	567	Farm	Rabbits, chickens	LT, ST
12	TI (LUG)	46.05588, 8.95031	443	Farm	Chickens, cattle	LT, ST
13	TI (LUG)	46.04391, 8.9411	443	Farm	Rabbits, chickens	LT, ST
14	TI (LUG)	46.00013, 8.92334	422	Farm	Rabbits, pigs, cattle, dogs	LT, ST
15	TI (LUG)	45.99488, 8.91427	276	Horse stable	Horses, dogs	LT, ST
16	TI (MEN)	45.86086, 9.00299	405	Farm	Rabbits, chickens, cattle, pigs	LT, ST
17	TI (MEN)	45.85826, 8.9958	364	Private breeder	Chickens, dogs	LT, ST
18	TI (MEN)	45.82656, 8.99956	277	Horse stable	Horses, dogs	LT, ST
19	TI (MEN)	45.82329, 9.0109	432	Private breeder	Rabbits, chickens	LT, ST
20	TI (MEN)	45.82089, 9.00587	447	Dog pension	Dogs, horses	LT, ST
21	BS	47.589258, 7.674204	385	Farm	Chickens	LT, ST
22	BS	47.574182, 7.623749	260	Farm	Chickens	LT, ST
23	BL	47.511855, 7.635353	404	Farm	Chickens, pigs	LT, ST
24	BL	47.495328, 7.545950	318	Farm	Chickens	ST
25	BL	47.500276, 7.568093	358	Farm	Chickens, horses	ST
26	BL	47.454196, 7.609626	384	Private breeder	Rabbits	ST
27	BL	47.418270, 7.503840	356	Private breeder	Rabbits	LT, ST

**Table 2:** Number and mean density (in brackets) of phlebotomine sand flies collected in Canton Ticino in 2022 and 2023. Mean density was calculated as the number of specimens collected divided by the sampling effort.

Site	Sampling Effort ^1^		*Ph. mascittii*		*Ph. neglectus*		*Ph. perniciosus*		*S. minuta*	
	2022	2023	2022	2023	2022	2023	2022	2023	2022	2023
1	7	0	0 (0)		0 (0)		1 (0.1)		0 (0)	
2	5	0	1 (0.2)		0 (0)		0 (0)		0 (0)	
3	6	0	1 (0.2)		0 (0)		0 (0)		5 (0.8)	
4	7	0	0 (0)		0 (0)		0 (0)		0 (0)	
5	7	0	0 (0)		0 (0)		0 (0)		0 (0)	
6	7	0	0 (0)		0 (0)		0 (0)		0 (0)	
7	6	0	0 (0)		0 (0)		0 (0)		0 (0)	
8	7	0	0 (0)		0 (0)		1 (0.1)		0 (0)	
9	7	0	3 (0.4)		0 (0)		5 (0.7)		0 (0)	
10	7	0	0 (0)		0 (0)		1 (0.1)		0 (0)	
11	7	0	0 (0)		0 (0)		0 (0)		0 (0)	
12	7	0	0 (0)		0 (0)		7 (1.0)		2 (0.3)	
13	7	0	1 (0.1)		0 (0)		0 (0)		0 (0)	
14	7	0	1 (0.1)		0 (0)		0 (0)		0 (0)	
15	7	0	0 (0)		0 (0)		1 (0.1)		0 (0)	
16	8	10	1 (0.1)	1 (0.1)	0 (0)	0 (0)	4 (0.5)	31 (3.1)	9 (1.1)	2 (0.2)
17	7	9	0 (0)	0 (0)	0 (0)	0 (0)	1 (0.1)	0 (0)	0 (0)	0 (0)
18	7	9	1 (0.1)	0 (0)	0 (0)	0 (0)	11 (1.6)	1 (0.1)	2 (0.3)	0 (0)
19	7	9	0 (0)	0 (0)	1 (0.1)	0 (0)	29 (4.1)	48 (5.3)	0 (0)	0 (0)
20	8	9	1 (0.1)	0 (0)	0 (0)	0 (0)	1 (0.1)	3 (0.3)	0 (0)	1 (0.1)
Total			10	1	1	0	62	83	18	3

^1^ Sampling effort: number of CO_2_-baited CDC light traps multiplied by the number of trapping nights.

**Table 3:** Results of the *Leishmania* sp. serosurvey (detection of antibodies) in dogs and cats. CI: Confidence Intervals.

		Dogs			Cats	
	n	%	95% CI	n	%	95% CI
Examined in this study	101			126		
Anti-*Leishmania* antibody positive	3	3	[0.6, 8.4]	2	1.6	[0.2, 5.6]
Anti-*Leishmania* antibody questionable	1	1	[0, 5.4]	-		
Anti-*Leishmania* antibody negative	97	96		124	98.4	

## Data Availability

All data generated or analyzed during this study are included in this published article.
